# Exploring the Relationship between Window View Quantity, Quality, and Ratings of Care in the Hospital

**DOI:** 10.3390/ijerph182010677

**Published:** 2021-10-12

**Authors:** Sahar Mihandoust, Anjali Joseph, Sara Kennedy, Piers MacNaughton, May Woo

**Affiliations:** 1 Center for Health Facilities Design and Testing, School of Architecture, College of Architecture, Arts and Humanities, Clemson University, Clemson, SC 29634, USA; smihand@clemson.edu (S.M.); skenne4@g.clemson.edu (S.K.); 2 View, Inc., Milpitas, CA 95035, USA; piers.macnaughton@view.com (P.M.); may.woo@view.com (M.W.)

**Keywords:** window views, nature views, hospital rating, quality of care, healthcare design, stress, patient satisfaction

## Abstract

Hospital ratings reflect patient satisfaction, consumer perception of care, and create the context for quality improvement in healthcare settings. Despite an abundance of studies on the health benefits of the presence and content of window views, there is a gap in research examining how these features may impact patient satisfaction and consumer perceptions of the quality of care received. A quantitative exploratory study collected data from 652 participants regarding their previous stay in the hospital, their perception of windows in their room, and their perception of their room, the hospital, and the quality of care received. On a scale of 0–10, participants with access to windows gave a 1-unit higher rating for the hospital. Access to window views from their bed provided a 1-unit increase, and having a view to green spaces resulted in a 2-unit increase in hospital ratings. Statistically significant results were also found for room ratings and care ratings. Windows in the patient rooms impact the key patient satisfaction measures and patient experience during the hospital stay. Patient room design, bed set up, and quantity and quality of window views may play an important role in shaping the patient’s experience.

## 1. Introduction

As healthcare becomes an increasingly consumer-driven industry, patient satisfaction has become extremely important for health systems. In the United States, patient satisfaction ratings such as those measured by the Hospital Consumer Assessment of Healthcare Providers and Systems (HCAHPS) not only provide institutions with a benchmark of their performance and perceived value for their customers, but also have deep financial implications as Medicare reimbursement is now inextricably linked to these ratings. Higher scores result in higher reimbursements, while low scores result in reduced funding from Medicare. Information regarding the patient’s perceptions about the care received and their overall satisfaction during their healthcare experience can incentivize positive change and quality improvement in healthcare [1]. Previous studies suggest that organizational interventions in a hospital, such as increasing nurse-staffing levels, may potentially improve patients’ experiences and perceptions. However, providing a higher quality of clinical care and a good experience for the patient also requires a focus on designing physical healthcare environments optimized for the patient experience [2,3].

One aspect of the physical environment that has been the focus of several studies is the presence of windows and the type of window views (nature versus urban view). Studies have explored the relationship between window presence, window views, and patient outcomes such as patient length of stay, patient satisfaction/preference, perception of pain, and patient stress [4,5,6,7,8]. However, patient satisfaction/preference, as measured in these studies, has been mainly explored through qualitative interviews [4,5]. This body of research suggests that windows in patient rooms may plausibly impact the patient’s experience during their hospital stay. However, there is a paucity of research exploring the quantitative impacts of patient room windows and window view characteristics on HCAHPS-relevant patient satisfaction measures and core metrics tracked by the hospitals and healthcare system administrators. Specifically, little is known about the impacts of the presence of a window, type of view, and size of window view on patients’ perceptions of their room, the quality of care received, and the hospital itself. These perceptions drive patient experience ratings such as HCAHPS scores. The financial incentive for an exceptional patient experience rating also lies in its impacts on hospital reputation, patient acquisition, and patient loyalty, which ultimately can drive hospital profitability [9]. Given the importance of improving patient experience and satisfaction, it is important to understand the role that windows can play.

The presence of a window view and natural light have often been studied together in previous research. Window views and natural light have shown to impact the perception of pain and length of stay in patients [6]. Chiu et al. (2017) investigated the effect of the presence of windows on ICU patient outcomes and found that patients with windowed rooms and daylight had shorter lengths of stay (4.8 days) compared to patients with windowless rooms (5.8 days). One study explored the relationship between the physical healthcare environment and patient satisfaction with the care unit and found that inpatients who perceived higher levels of physical and social environment quality with regards to comfort, orientations, view, and daylight had higher patient satisfaction levels [10].

Window views of nature have shown a strong relationship with shorter length of stay [11], reduced perception of pain [4], relaxation and positive emotions [12], and patient satisfaction with their view [5]. A study of ICU patients concluded that patients with views of nature had a reduced length of stay compared to patients with urban views [11]. A study of surgical patients found that those with a window view of trees had fewer negative comments from nurses during evaluation, took fewer doses of pain medications, and showed lower scores for minor postsurgical complications [4]. According to another study, patients were unsatisfied with hospital rooms without windows or with a poor view and noted that the window conditions were ideal if the view had elements of trees, other natural elements, or the sky [5]. A study of rehabilitation center residents explored the impact of view through windows and indoor plants on recovery and found a strong relationship between the type of window view and view pleasantness. Patients with a panoramic window view to nature found their view calming, and those with a blocked view to the outdoors were dissatisfied with the view [12]. Participants in a study by Gharaveis et al. (2016) emphasized that the view content was important to them, with view of nature and other people’s activities noted as their preferred type of view [8]. Lyendo et al. (2016) explored the effects of landscape views on hospital occupants and found aesthetic appreciation and improved health and well-being from patients. This study also supported the concept that exposure to nature promotes positive feelings such as calmness and reduced anxiety [13]. While previous research has evaluated aspects of satisfaction with the window view qualitatively, none of these studies examined the relationship between window presence and view type with HCAHPS-relevant patient satisfaction measures, including patient satisfaction with their room, hospital, or quality of care received.

Window size can also impact patient satisfaction with the room and other health outcomes. Verderber and Reuman (1987) found that a group of patients were negatively affected by windows with sills that were too high (above the floor, at 48”). These patient groups included patients with mobility problems, impaired vision, paralyzed patients, and disabled patients who could not access the view outside the window due to windowsill height [14]. Additionally, small-sized windows in the patient rooms were found to be equally unsatisfactory as rooms with no windows [5]. Other research suggested that patient satisfaction was independent of the window size and shape [8]. In a follow-up study, Gharaveis et al. (2020) interviewed care providers in an ICU and found that staff preferred larger windows in the unit to allow more daylight into the patient rooms and to provide better views to the outside for the patient [15].

Window location and access to view from the patient bed have also been deemed important in previous studies. One study found that only 47% of patients preferred to have a direct view outside from the window next to their bed, and privacy and lighting control were more important. Although most patients did not want their window directly next to their bed, more than half changed their position within their room to better view outside or move away from light disturbances, gaze, and unwanted lighting reflections [8]. Clinical staff has also reflected on these patient desires, reporting that patients likely prefer to have windows next to their bed to have a view outside, but not in direct sunlight. In this study, more than half of the participants thought having a window in front of the bed or near the foot of the bed would be most effective [15]. Additionally, one study found that patients were challenged by poorly positioned windows and could not maintain a connection with the outside [14].

The presence of windows and the type of view have impacted different aspects of patient satisfaction, health outcomes, and overall perception of the environment. However, more research is needed to quantify the impact of window presence, window view content, and window size on patient experience as measured in ratings and their perception of healing and stress-relieving qualities of patient rooms. This research aims to further investigate the relationship between the window view quantity and quality in patient rooms and patient experience as described by the hospital, care, and room ratings. The key research questions addressed in this study are as follows:

1. What is the relationship between the presence/absence of windows and patient experience as measured by the hospital, care, and room ratings?
2. What is the relationship between the presence/absence of access to view from the position on the bed and patient experience as measured by the hospital, care, and room ratings?
3. What is the relationship between view content and patient experience as measured by the hospital, care, and room ratings?

## 2. Materials and Methods

### 2.1. Data Collection

The study collected data using a nationwide online survey (25 November–15 December 2020) of patients who received care within the past year in hospitals across the U.S. The survey included 174 questions and involved different sections on the following: (a) patient demographics and clinical information (age, gender, race, education, length of stay, reason for stay, admitting unit, and admission reason); (b) presence of windows in the patient room, window blinds position, presence of a view from the patient’s bed, size of windows, need for more windows, and view content in the patient’s room; (c) retrospective ratings of the hospital, patient room, and care (in the hospital and the patient room where the patient stayed on their recent visit), as well as the perception of patient room and hospital environment in relieving stress and fostering healing and recovery; and (d) a section on comparing a set of patient room images for different hospital ratings. Study questions were created by two study experts based on existing literature and were revised after receiving feedback from two other experts. Survey questions were pilot tested and revised prior to data collection. This study received approval from the Clemson University Institutional Review Board and was distributed via Qualtrics. A total of 1901 responses were collected, of which 652 fit the inclusion criteria of being a U.S. resident aged 18 or over, having spent at least one night in an inpatient stay in the past 12 months, and having passed the data quality checks, which included removing duplicate responses, responses that were completed under the minimum survey duration threshold, and straight-lined or patterned responses for matrix questions.

This study only examined the responses to the first three sets of questions: the first set of questions that collected data on patient demographics and clinical information; the second set, which included six questions on the presence of windows in the patient rooms, window blinds position, the presence of view from the patient’s bed, size of windows, need for more windows, and view content in the patient’s room; and the third set, which included five questions on hospital rating, room rating, and quality of care rating, as well as the perception of the hospital and the patient’s room in response to patient stress, healing, and recovery.

### 2.2. Study Measures

#### 2.2.1. Independent Variables

The presence of windows and the view from the patient’s position on their bed were determined by questions inquiring: “During your stay, did your room have a window?” and “In this patient room, were you able to see the view outside the window from your position on the bed?” The responses to these questions were binary (yes, no). Window size and the need for more windows were determined by prompts to “Indicate your level of agreement with this statement: This patient room had large windows” and “This patient room needed more windows.” The provided category of responses included “totally agree, agree, neutral, disagree, and totally disagree.” The response categories “totally agree” and “agree”, and “disagree” and “totally disagree” were merged into “agree” and “disagree”, respectively, to facilitate data analysis. Window view content was determined by “Indicate your level of agreement with this statement: In this patient room you could see green spaces from the windows.” Similar to the two previous items, the initial response categories included “totally agree, agree, neutral, disagree, and totally disagree” and were merged into three categories for analysis: “agree”, “neutral”, and “disagree.”

#### 2.2.2. Outcome Variables

Ratings of the hospital were determined by asking the participants “How would you rate this hospital during your stay on a scale of 0–10?”; the rating of care was determined by asking “How would you rate the overall quality of care that you received during your stay on a scale of 0–10?”; the rating of the patient’s room was determined by asking “How would you rate the satisfaction with your patient room during your stay on a scale of 0–10?”. The definition for the scale of rating provided to the patient was “where 0 was the worst/lowest possible outcome, and 10 was the best/highest possible outcome”. These question types and rating scales were adopted to parallel the analogous questions used in the standard HCAHPS survey. The two qualitative variables, “room relieving stress” and “hospital fostering healing,” were also measured by asking participants to indicate their “level of agreement” with the following statements: “The hospital room environment helped in relieving my stress” and “The hospital provided a care environment that fostered my healing and recovery”. The initial category of responses for these questions included “totally agree, agree, neutral, disagree, and totally disagree”, and were merged into three categories for analysis: “agree”, “neutral”, and “disagree”.

### 2.3. Analysis

Different criteria were used for data inclusion while addressing the study research questions. For comparing the study outcomes with regards to presence or absence of windows in patient rooms, data from all participants were utilized (*N* = 652). When comparing the group who had access to window views from their position on the bed versus not having views, data from the participants who reported no windows during their stay and from participants who reported “Don’t know/Can’t remember” regarding their view condition were excluded, resulting in the inclusion of 513 responses. For comparing the outcomes with respect to window size and the need for more windows, only the responses for the participants with windows and who selected “agree” and “disagree” for the questions regarding window size and the need for more windows were included for the analysis (*N* = 433 and *N* = 413, respectively). Finally, for comparing the participant responses with respect to access to green spaces from their window view, responses for participants with no windows and participants who had their blinds completely shut were eliminated, and responses from participants who selected “agree” and “disagree” for the questions regarding access to green space (in their window view) were included for the analysis (*N* = 348).

The Mann–Whitney U test was conducted to compare the quantitative results (ratings of hospital, care, and room) following groups for the non-parametric data distribution with unequal group sizes: participants in rooms with windows vs. no windows, view from the position on the bed vs. no view from the position on the bed, view to green spaces vs. no view to green spaces. Additional analysis including the Mann–Whitney U test was conducted and compared the participant ratings of those who agreed or disagreed with having large windows or the need for more windows separately. The Chi-square test of independence was performed to examine the relationship between presence of windows, access to views, access to green spaces, and the perception of healing and recovery in the hospital and an environment relieving stress in the patient’s room.

## 3. Results

### 3.1. Participants

The majority of the participants in this study were below 40 years old (70%). Approximately half the study participants were male. Most of the study participants had a college education (78%) (Table 1). The majority of the participants stayed in the hospital for less than a week (84%). Participants reported an inpatient stay for various reasons, including childbirth, injury, medical, mental health, surgery, and others. Approximately a quarter of the participants were admitted to either the emergency department (24.4%) or the medical surgical unit (25.3%) (Table 2).

Rating of satisfaction with the room, quality of care, and hospital were highly correlated (*p* < 0.001) for the participants who responded to these questions. The correlation between hospital rating and quality of care rating, hospital rating and satisfaction with room rating, and quality of care rating and satisfaction with room ratings were r = 0.86, r = 0.80, and r = 0.79, respectively, (*p* < 0.001).

### 3.2. Presence of Windows

**Figure 1:**
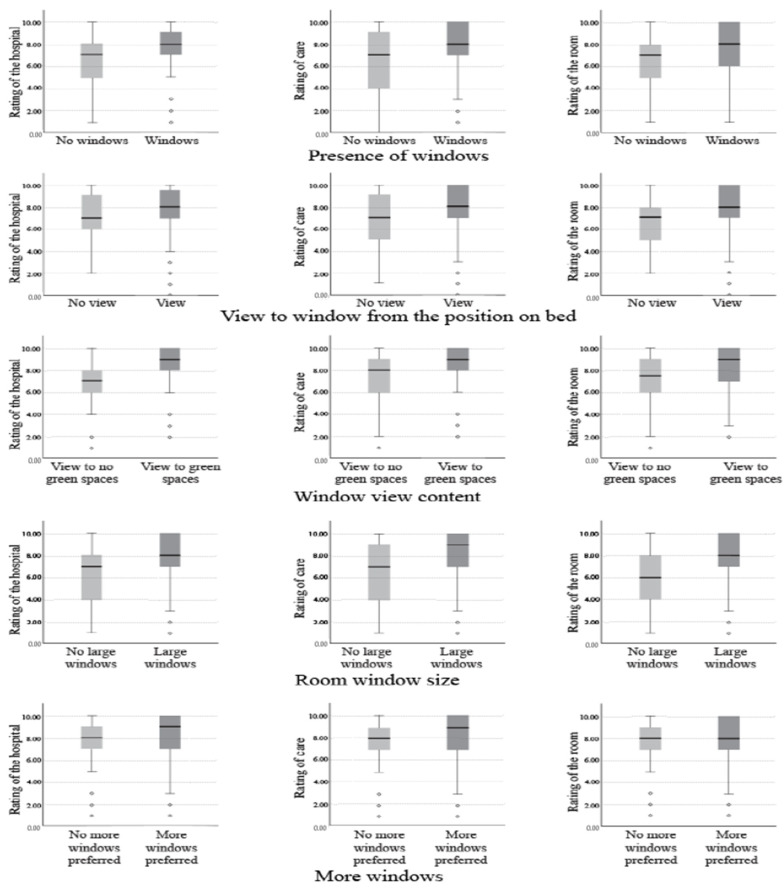
The distribution of scores for windows, views, window view content, window size, and quantity.

Participants who received care in the patient room with windows (*N*_windows_ = 579, median = 8) rated the hospital higher than participants in the patient room with no windows (*N*_no windows_ = 73, median = 7). A Mann–Whitney test indicated this difference was statistically significant (U = 14639, z = −0.34, *p* < 0.001). The participants in the patient rooms with windows also rated the quality of care and satisfaction with the patient room as significantly higher than the participants in the patient room with no windows (U = 15031, z = −4.09, and *p* < 0.001 and U = 15287, z = −3.9, and *p* < 0.001, respectively). Table 3 summarizes the results of the Mann–Whitney U tests, and Figure 1 displays the distribution of scores for windows, views, window view content, window size, and quantity.

**Figure 2:**
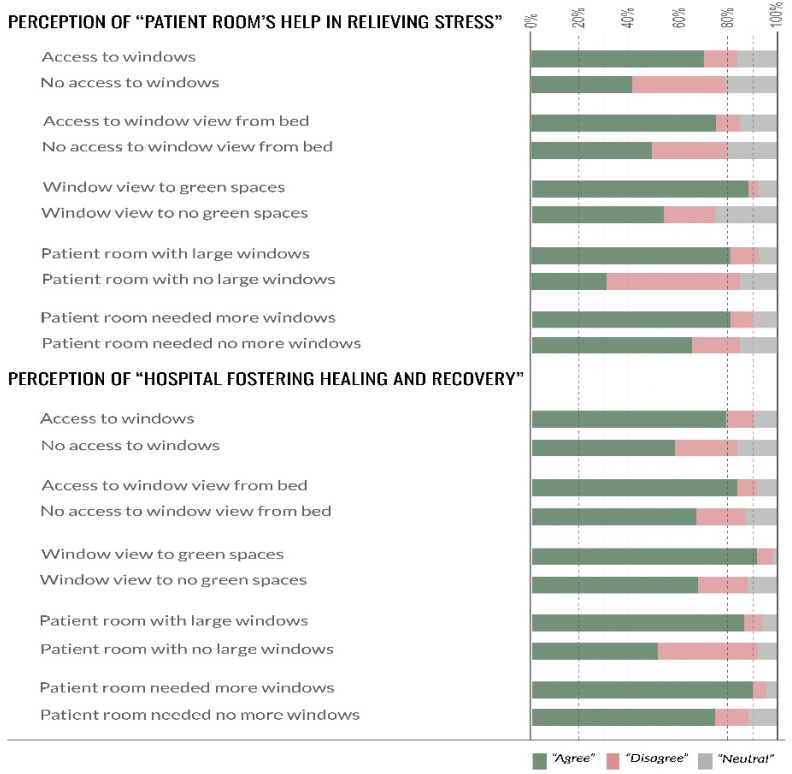
Chi-square test comparisons for “patient room relieving stress” and “hospital fostering healing” across participants with windows/no windows, view from bed/no views, access to green views/no green views, window size, and need for more windows.

The relationship between the presence or absence of windows and the perception about the room environment helping to relieve the patient’s stress was significant (X2 (1, *N* = 648) = 35.26, *p* < 0.001). However, the effect size was small (0.23). Participants in the room with windows were more likely to perceive the room environment helping to relieve their stress. A higher percentage of the participants in the patient rooms with windows (70%) perceived the room environment to help in relieving their stress in comparison to only 40% of the participants who stayed in rooms with no windows (Figure 2). The relationship between the presence or absence of windows and perception about the hospital care environment fostering healing and recovery was significant (X2 (1, *N* = 648) = 17.45, *p* < 0.001). However, the effect size was small (0.16). Participants in the room with windows were more likely to perceive that the hospital care environment fostered their healing and recovery. A higher percentage of the participants in the patient rooms with windows (80%) perceived the hospital care environment to foster their healing and recovery compared to the participants who stayed in rooms with no windows (58%) (Figure 2).

### 3.3. View from Bedside

Among participants who were in rooms with windows, participants who were able to see the view outside their window from their position on the bed (*N*_view_ = 444, median = 8) rated the hospital higher than participants in the patient room with no view from their position on the bed (*N*_no view_ = 69, median = 7). The Mann–Whitney test indicated this difference was statistically significant (U = 11416, z = −3.46, and *p* = 0.001). The participants in the patient rooms with views also rated the quality of care and satisfaction with the patient room significantly higher than the participants in the patient rooms with no views (U = 11019, z = −3.82, and *p* < 0.001 and U = 9883, z = −4.82, and *p* < 0.001, respectively) (Table 3, Figure 1).

The relationship between access/no access to a view outside from the position on the bed and the perception of room environment helping in relieving the patient’s stress was significant *(X*^2^ (1, *N* = 511) = 10.08, *p* < 0.001). However, the effect size was small (0.22). A higher percentage of the participants (75%) in the patient rooms with views from bed perceived that the room environment helped relieve their stress compared to a similar response from only 49% of the participants who stayed in rooms with no views. The relationship between access/no access to view outside from the position on the bed and the perception that the hospital care environment fostered healing and recovery was significant *(X*^2^ (1, *N* = 511) = 25.92, *p* < 0.001). However, the effect size was small (0.14). Participants who had access to views outside from their position on the bed were more likely to perceive that the hospital care environment fostered their healing and recovery. A higher percentage (83%) of the participants in the patient rooms with views from the bed perceived that the hospital care environment fostered their healing and recovery compared to 68% of the participants who stayed in rooms with no view from their bed (Figure 2).

### 3.4. View Content

Participants who received care in patient rooms with windows and agreed that they viewed green spaces (*N*_green spaces_ = 268, median = 9) outside their window rated the hospital higher than participants who disagreed with viewing green spaces outside their window (*N*_no green spaces_ = 80, median = 7). A Mann–Whitney test indicated this difference was statistically significant (U = 6117, z = −5.987, *p* < 0.001). Participants in the patient rooms with views to green spaces (median = 9) rated the quality of care significantly higher than the participants in the patient room with view to no green spaces (median = 8) (U = 6601, z = −5.395, *p* < 0.001). Similarly, these participants also rated the satisfaction with the room significantly higher (median = 9) than the participants in the patient room with view to no green spaces (median = 7.5) (U = 6909, z = −4.98, *p* < 0.001) (Table 3, Figure 1).

The relationship between view content (green spaces vs. no green spaces) and the perception of the room environment in helping to relieve the patient’s stress was significant *(X*^2^ (2, *N* = 348) = 53.81, *p* < 0.001), and the effect size was moderate (0.39). A higher percentage of the participants (90%) that reported viewing green spaces from their windows perceived that the room environment helped in relieving their stress, while only 53% of the participants who disagreed with having a view to green spaces out of their patient room window felt the same way. The relationship between view content (green spaces vs. no green spaces) and the perception about that the hospital care environment fostering healing and recovery was significant *(X*^2^ (2, *N* = 348) = 32.62, *p* < 0.001), and the effect size was moderate (0.30). A higher percentage of the participants in the patient rooms with views to green spaces (92%) perceived that the hospital care environment fostered their healing and recovery compared to 68% of the participants without views to green spaces out of their patient room window (Figure 2).

### 3.5. Window Size

Participants who received care in the patient room with windows and agreed with having large windows rated the hospital one score higher than the participants who disagreed with having large windows (*p* < 0.001). The group that agreed with having larger windows also rated the quality of care and satisfaction with the patient room two points higher than the group who disagreed with having large windows (*p* < 0.001) (Table 3, Figure 1).

The relationship between window size (large vs. not large) and the perception of the room environment in helping to relieve the patient’s stress was significant *(X*^2^ (2, *N* = 433) = 82.15, *p* < 0.001), and the effect size was moderate (0.43). A higher percentage of the participants in the patient room with large windows (80%) perceived the room environment to help relieve their stress compared to only 32% of the participants who disagreed that they had large windows in their patient room. The relationship between window size (large vs. not large) and the perception about the hospital care environment fostering their healing and recovery was significant *(X*^2^ (2, *N* = 433) = 48.9, *p* < 0.001), and the effect size was moderate (0.33). A higher percentage (87%) of the participants who reported having larger windows in their patient rooms perceived the hospital environment to foster their healing and recovery compared to only 52% of the participants who stayed in rooms with no large windows (Figure 2)**.**

## 4. Discussion

This study collected and compared ratings of the hospital, care, and the patient room to measure patient experience and satisfaction outcomes related to HCAHPS with regards to presence of windows, views, and view content. The presence of windows, access to views from the bedside, and views to green spaces all had a significant impact on the satisfaction outcomes related to HCAHPS ratings of the hospital, quality of care, and satisfaction with the room. Participants with access to windows or access to views from bedside gave a 1-unit higher rating for the hospital, care, and patient room compared to participants in rooms that lacked these features. Participants with access to green views rated the hospital, care, and patient room 1 to 2 units higher in comparison to participants who did not have access to green views. Patient satisfaction as measured by HCAHPS-related outcomes indicates a positive patient experience, increased utilization, patient loyalty, and provides a benchmark for hospital’s clinical performance [16,17]. Higher HCAHPS-related patient satisfaction scores are also related to higher Medicare reimbursement for the hospital.

Utilization of HCAHPS survey tools to measure patient satisfaction is a relatively recent development with significant financial impacts for healthcare organizations. Understanding environmental predictors of high satisfaction scores in the hospital setting can support healthcare administrators in allocating resources to critical environmental features that could improve patient satisfaction [18]. Previous studies investigating the impact of built environment factors on HCAHPS-related satisfaction measures have primarily focused on auditory aspects of the environment [19,20,21]. This study makes a significant contribution to the literature by examining the relationship between HCAHPS-related patient satisfaction measures and window-related variables. Additionally, previous studies have only explored the impact of windows and view quality on patient satisfaction through qualitative methods or as a bundle combined with other environmental features. The quantitative data from a large national sample in this study adds to the body of research surrounding the impact of the physical environment on patient satisfaction in healthcare settings. Previous studies have identified the significant role of the physical setting of the hospital and the patient room on patient satisfaction with care or the hospital, emphasizing environmental satisfaction as a significant predictor of satisfaction [3,22,23,24]. However, the impact of individual elements such as the presence of windows and location of bed vis-à-vis windows on patient satisfaction has not been studied before. Findings from this study demonstrate that aspects of the physical environment of the patient room, specifically the presence and characteristics of windows in the room, are related to a range of HCAHPS-related patient satisfaction measures. This study found that both presence of windows and the position of the bed in relation the windows clearly impacted patient ratings of the hospital, quality of care, and patient room. Ratings of the hospital, care, and patient room were significantly higher when the participants had windows versus no windows. The participants in the rooms with windows also rated the room as relieving stress and the hospital fostering healing and recovery significantly better than the participants in the rooms with no windows. Participant perception regarding the hospital environment fostering healing and recovery aligns with previous studies suggesting that the patients in rooms with windows had a shorter length of stay [6]. This study also found that participants who had views to outside from their position on the bed had significantly higher ratings of the hospital, care, and patient room compared to the group that had windows but no view to outside from the position on the bed. A proper layout of the patient room, position of the bed vis-à-vis the window, and a proper windowsill height enabling the patient lying in bed to access the window view led to better HCAHPS-related patient satisfaction scores. Hospital designers and administrators should consider these factors when designing new facilities or renovating existing facilities.

The view content was an important factor impacting the rating of the hospital, care, and patient room and also the perception of the room for relieving stress and the perception of the hospital for fostering healing and recovery. These findings align with previous research on the impact of nature views on stress recovery for patients [25]. Participants who reported viewing green spaces had significantly higher scores and ratings for all study outcomes compared to the group who did not report viewing green spaces. Additionally, the effect size of the view content on the perception of the room environment helping in relieving the patient’s stress and hospital care environment in fostering healing and recovery was moderate, while the presence of windows or access to views, had only a small effect size on these two outcomes. Therefore, the content of the view was the key factor determining not only the rating of the hospital, care, and patient room, but also patients’ perception of the room in relieving stress and supporting recovery in the hospital. While many studies only study the window presence alongside daylight (not studying the views), the results of this study suggest that studying the access to windows or views without exploring the view content may leave out a significant portion of the explanatory information. Providing a window with a view to nature or greenery may not always be feasible when designing patient rooms, but designers and architects should attempt to provide views of nature through their approach to site layout, landscaping, and campus planning. It is also worthwhile to mention that patient room windows may sometimes be the only major source of views to the outdoors for clinical staff in the hospital [26]. Therefore, providing window views to nature could also improve well-being for the clinicians and ameliorate negative workload-related outcomes such as burnout [27], thus impacting both clinician and patient satisfaction.

Window size was another important factor influencing the five study outcomes. However, this study suggests that the impact of window size on patient ratings of the hospital, care, and patient room may also be dependent on the window view content. Study findings regarding the impact of bigger window view size and view content on patient satisfaction align with previous research that indicated medium-sized and highly humanized hospital units scored significantly higher for perceived hospital environment quality indicators; the large size of windows and views to nature were among the environmental determinants for medium-sized and highly humanized units [28].

This study recruited a large sample of 652 participants from across the United States, providing a more accurate representation of the patient experience during a patient’s hospital stay and a greater precision and power for the findings of the study. The national sample also makes the findings more generalizable than a study design focusing on a single hospital or healthcare organization.

## 5. Limitations

The majority of the participants in this survey study were below 40 years old, and thus a younger population compared to typical hospital demographics. Older participants may have different levels of acuity and reactions to daylight and views than a younger population. Additionally, many participants had shorter hospital stays than might be expected in the typical hospital inpatient. When comparing the presence or absence of windows, bedside view vs. windows, and window view content, some subgroups had small sample sizes that limited statistical significance [29]. Another limitation of the study refers to the lack of survey questions regarding lighting comfort. The inclusion of measures related to the perception of lighting comfort in the patient rooms could provide an additional level of understanding regarding the impacts of windows in healthcare facilities. Finally, participant responses were based on recall of the previous stay up to 12 months prior to the survey, and there is the potential for recall bias compared to data collected during an actual hospital stay.

## 6. Conclusions

Patients who perceived views to green spaces from their rooms reported higher hospital, care, and room ratings in comparison to patients who viewed non-green spaces, had no views, or had no windows. Window access, size, and view quality in the patient rooms impacted the HCAHPS-related patient satisfaction measures and patient experience during the hospital stay. Based on these findings, designers and architects should consider the following: (1) aspects of the patient room design that will increase access to views, such as window size, room layout, and patient bed location; and (2) aspects of the site design that will increase the quality of the views, such as building orientation, landscaping, and campus planning.

## Figures and Tables

**Table 1:** Participant demographic information.

Demographics	*N*	Percentage
Age		
18–30	213	32.67%
31–40	237	36.35%
41–50	137	21.01%
51–60	43	6.60%
Above 60	22	3.37%
Gender		
Female	322	49.39%
Male	324	49.69%
Non-binary	5	0.77%
Prefer not to answer	1	0.15%
Education		
High-school degree	136	20.86%
Some college	153	23.47%
Higher education	358	54.91%
Prefer not to answer	5	0.77%
Race		
White	477	73.16%
Asian	26	3.99%
Black or African American	108	16.56%
other	37	5.67%
Prefer not to answer	4	0.61%

**Table 2:** Clinical information for patients’ hospital visits.

Clinical Information	*N*	Percentage
Admission unit		
Coronary care and cardiothoracic unit (CCU)	34	5.21%
Emergency department (ED)	159	24.39%
Intensive care unit (ICU)	98	15.03%
Labor and delivery unit	75	11.50%
Rehabilitation ward	44	6.75%
Medical-surgical unit	165	25.31%
Do not know	53	8.13%
Other	24	3.68%
Admission Reason		
Childbirth	73	11.20%
Injury	140	21.47%
Medical	223	34.20%
Mental health/substance abuse	48	7.36%
Surgery	137	21.01%
Other	31	4.75%
Length of stay		
1–2 days	354	54.29%
3–7 days	197	30.21%
1–2 weeks	62	9.51%
2 weeks–1 month	18	2.76%
More than 1 month	21	3.22%

**Table 3:** Results of the Mann–Whitney U test for windows, view from bed, view content, window size, and quantity.

**Ratings of the hospital (1), care (2), and patient room (3)**	**Mean (SD)**	**Median**	**Mean (SD)**	**Median**	**U**	**Z**	***p***	**Cohen’s d**
	**Windows (*N* = 579)**		**No windows (*N* = 73)**					
(1)	7.61 (2.14)	8	6.23 (2.66)	7	14639.5	−4.34	0	−0.17
(2)	7.78 (2.15)	8	6.34 (2.91)	7	15031.5	−4.09	0	−0.16
(3)	7.62 (2.20)	8	6.23 (2.91)	7	15287	−3.911	0	−0.15
	**View to outdoor (*N* = 444)**		**No view (*N* = 69)**					
(1)	7.81 (2.05)	8	6.80 (2.42)	7	11416.5	−3.461	0.001	−0.15
(2)	7.98 (2.05)	8	6.86 (2.46)	7	11019.5	−3.824	0	−0.17
(3)	7.83 (2.14)	8	6.54 (2.19)	7	9883.5	−4.823	0	−0.21
	**View—green space (*N* = 269)**		**View—no green spaces (*N* = 80)**					
(1)	8.37 (1.73)	9	6.42 (2.17)	7	6117.5	−5.987	0	−0.32
(2)	8.56 (1.66)	9	7.28 (2.14)	8	6601.5	−5.395	0	−0.29
(3)	8.14 (1.75)	9	7.03 (2.37)	7.5	6909	−4.982	0	−0.27
	**Large windows (*N* = 388)**		**No large windows (*N* = 46)**					
(1)	8.04 (1.89)	8	6.41 (2.67)	7	5609	−4.199	0	−0.2
(2)	8.21 (1.86)	9	6.57 (2.67)	7	5766.5	−4.015	0	−0.19
(3)	8.11 (1.88)	8	6.00 (2.65)	6	4746	−5.302	0	−0.25
	**More windows (*N* = 254)**		**No more windows (*N* = 159)**					
(1)	8.00 (2.10)	9	7.40 (2.12)	8	16242	−3.407	0.001	−0.17
(2)	8.14 (2.04)	9	7.63 (2.21)	8	17195	−2.596	0.009	−0.13
(3)	7.86 (2.15)	8	7.61 (2.20)	8	18740	−1.254	0.021	−0.06

## Data Availability

The data that support the findings of this study are available from the authors upon reasonable request.
